# Influence of the CoViD-19 Pandemic on Mental Workload and Burnout of Fashion Retailing Workers in Spain

**DOI:** 10.3390/ijerph18030983

**Published:** 2021-01-22

**Authors:** Ana María Rodríguez-López, Susana Rubio-Valdehita, Eva María Díaz-Ramiro

**Affiliations:** Faculty of Psychology, Campus de Somosaguas, University Complutense of Madrid, 20223 Madrid, Spain; anrodr17@ucm.es (A.M.R.-L.); ediazram@ucm.es (E.M.D.-R.)

**Keywords:** mental workload, burnout, CoViD-19, pandemic, fashion retailing sector, Spain

## Abstract

This study analyzed the levels of mental workload and the presence of burnout on a sample of fashion retailing workers from Spain and its relationship with the current CoViD-19 (Coronavirus disease-19) pandemic. We established a cross-sectional design. Participants (*n* = 360) answered an online survey including questions about sociodemographic data, perception of CoViD-19, CarMen-Q questionnaire (workload), and MBI (burnout syndrome). The survey campaign took place in October and November 2020. The results showed that participants exhibited deep concern about the CoViD-19 pandemic and its influence in the workplace. Although the mental workload was near the middle point of the scale, participants showed moderate to high burnout levels, revealing that the sample was at risk of experiencing higher burnout levels over time as the pandemic and associated economic crisis continued. The multiple regression analysis results indicated that environmental changes, work overload, somatic symptoms, insomnia, negative job expectations, and uncertainty constituted significant mental workload predictors. Insomnia, somatic symptoms, and negative job expectations constituted significant predictors for burnout. Differences between job positions and genders in mental workload and burnout were found. In conclusion, the uncertainty at work derived from the CoViD-19 pandemic harms fashion retailing workers’ psychological well-being in Spain.

## 1. Introduction

Today, a “healthy company” does not focus solely and exclusively on obtaining satisfied customers but must also ensure that the people who make up the organization itself, that is, its employees, enjoy healthy working conditions. Due to this new perspective, companies are varying their course by changing human resources management, which now focuses on the psychosocial health of the people who make up the workforce to obtain beneficial results. It is necessary to consider this last point, since it has been possible to verify the correlation that “good health” has with the social and economic benefits that the organization can have [1].

Globalization’s effects are the constant change of the workplace, which has forced organizations to adapt to the changing nature of the work context, the development of information and technological communication, and demographic changes. That is why in Europe, corporate social responsibility (CSR) has developed on a large scale in the last decade. There are several definitions to understand how CSR works within organizations [2]. Some authors define it as a concept by which companies integrate social and environmental concerns in their business strategies. Other authors expand this concept with the idea that there are two dimensions: internal and external. The first dimension deals with practices regarding the employees’ concerns about their well-being and health. On the other hand, the external dimension refers to the company’s social environment, such as suppliers.

In December 2019, a cluster of 27 pneumonia cases emerged in Wuhan (Hubei, China), with a common exposure to a wholesale market for seafood, fish, and live animals. On 7 January 2020, Chinese authorities identified as the causative agent of this outbreak a new virus of the Coronaviridae family, which was later named SARS-CoV-2. Chinese authorities shared the genetic sequence on 12 January. On 11 February 2020, the WHO (World Health Organization) called the disease CoViD-19 by consensus. The Emergency Committee of the Health Regulations International (ECHRI) declared the outbreak a Public Health Emergency of Major International (PHEMI) at its meeting on 30 January 2020. Subsequently, the WHO recognized it as a global pandemic on 11 March 2020 [3]. 

In mid-March 2020, the Spanish government declared a state of national emergency due to the health crisis. To control the high number of infections, the government decreed compulsory home confinement. In the health field, all nonpriority medical acts were limited, and urgent activity was maintained. To monitor the number of cases, the Spanish government established a reporting system of all confirmed CoViD-19 cases in each Autonomous Community (AC) through an epidemiological survey. The survey includes clinical-epidemiological information approved by the Report on Alerts and Preparedness and Response Plans and the National Epidemiological Surveillance Network. Each AC must continually update the survey information [4].

Italy was the first European country to exhibit alarming data on the virus’s lack of control [5]. However, the Italian infection curve was only a week or two ahead of developments in Austria, Spain, Switzerland, France, Germany, and the United Kingdom. Thus, Italy and Spain were the first and worst affected countries in Europe’s first wave of CoViD-19. According to the National Institute of Statistics [6], in the first wave caused by CoViD-19 in Spain (between January and May), 45,684 people died from this disease. In addition to the 45,684 deaths due to CoViD-19, doctors certified another 4218 cases in which the cause of death was not directly the disease, although the virus contributed as a comorbidity. 

In June 2020, the state of national emergency in Spain ended with devastating consequences for the Spanish economy. During the lockdown, the Spanish GDP (Gross Domestic Product) dropped 22.1%. The International Monetary Fund estimated a 12.8% drop in Spain’s GDP by the end of 2020 and a 6.3% recovery in 2021. On the other hand, the Organisation for Economic Co-operation and Development estimated an 11.1–14.4% drop in Spain’s GDP and a 5–7.5% recovery in 2021. 71% of Spanish entrepreneurs described their economic situation as “bad” or “very bad” [7].

Today, the entire world is facing a second wave of infections by CoViD-19. Data from Eurostat and national statistical agencies (Scotland: NRS, Northern Ireland: NISRA, Germany: Destatis) show that deaths by CoViD-19 in the second wave are higher than in the first wave. Western European countries such as Italy and Spain, which were the most affected on the first wave, are suffering nearly as badly. Central and Eastern Europe, which were barely affected by the first wave, now exhibit alarming spikes in cases and deaths [8]. According to the European Centre for Disease Prevention and Control, as of week 52, 2020, the EU/EEA reported 17,348,389 cases and 427.798 deaths, with France, United Kingdom, Italy, Spain, Germany, and Poland being the most affected countries. In Spain, the sum of cases reached 18,794,13 and the sum of deaths 50,122. However, the number of deaths in Spain’s second wave is lower than in the first. Multiple reasons could explain this decrease in the number of deaths: highest mortality of the first wave, knowledge acquired about the virus, better management of time and sanitary spaces, improved hospital equipment, and increment of diagnostic resources.

Even though the Spanish GDP increased by 16.5% after the end of the state of national emergency, the second wave’s appearance has led to maintaining these pessimistic predictions. Thus, due to this second wave, the Organisation for Economic Co-operation and Development estimated a 12.8% drop in Spain’s GDP by December with a higher impact on the retail sector and the tourism sector. By now, 33,000 businesses have disappeared, and large companies have not managed to trace their sales [9].

Over the last decade, fashion retailing in Spain has significantly increased its value in the market, reflected in the intensification in job offers, approximately 132,000, and its value-added by 4.1% in the manufacturing industry [10]. In 2019, fashion retailing suffered a 20% drop in revenue compared to 2007, and experts estimate an additional 35–40% drop in 2020, according to an EY (Ernst & Young) and The Boston Consulting Group study [11]. The impact caused by the CoViD-19 (Coronavirus disease-19) pandemic on fashion retailing is immediate: more than 65,000 people from fashion retailing will lose their jobs due to the closure of sales points and the disappearance of smaller businesses [12]. The Spanish government allowed shops and shopping centers to open under multiple restrictions to reactivate fashion retail economically. Restrictions are the following [13]:

- Protective measures against the virus: mandatory hygiene measures that workers must carry out (i.e., use of a mask, interpersonal distance of 2 m, hygiene and disinfection of hands and personal objects).
- General hygiene and protection measures: mandatory hygiene measures that establishments must carry out (i.e., the existence of protective material for the entire staff, protocol in case a worker tests positive for coronavirus or maintains contact with someone infected, cleaning protocol of the uniform, cleaning protocol of the establishment).
- Specific measures: organizational measures (i.e., entry and exit to the workplace in a staggered manner, limit the number of workers in the workplace, daily monitoring of body temperature on entry and exit from work, facilitate online sales, the establishment of limited capacity in stores and shopping centers).

On the other hand, the governments of each AC have also established specific restrictions: Madrid, Murcia, Cantabria, Extremadura, and Cataluña establish a perimeter closure by municipalities which prevents travel to stores or shopping centers outside the municipality of residence; Andalucía, Asturias, Galicia, and Baleares established the mandatory closure of all stores and shopping centers at 6:00 p.m.; Castilla-La Mancha established the temporary closure of all stores and shopping centers, Castilla y León, Extremadura, Navarra, Aragón, and Cataluña reduced the capacity of stores and shopping centers by 30%. Ceuta y Melilla, Comunidad Valenciana, La Rioja, País Vasco, and Canarias have not implemented specific measures [14].

Thus, due to the economic difficulties of recent decades and the emergence of large international competitors, added to the recent pandemic caused by the CoViD-19 virus, have led to a tightening of the working conditions of workers in this sector and, consequently, to increase the prevalence of occupational diseases such as burnout related to mental workload [15,16]. Mental workload is a multidimensional concept that implies the difference between a job’s cognitive demands and an employee’s cognitive skills [17]. Mental workload is task- and person-specific and involves motivation, individual capacities, and performance [18].

On the other hand, the most accepted definition of burnout describes it as a “syndrome of emotional exhaustion and cynicism that frequently occurs among individuals who do people work of some kind” [19]. Thus, several studies have analyzed the effects of stress on professional selling employees [20,21], showing that stress is inherent to this sector due to its client-oriented nature. This type of stress can lead to adverse physical and psychological effects as burnout [22]. 

Recent studies have estimated that the psychological impact caused by the CoViD-19 pandemic is like that of a catastrophe: 1 in 5 people will manifest depressive and anxious symptoms [22]. Over the last year, numerous studies have revealed these consequences in health sector workers worldwide. Khasne et al. [23] showed that more than half (52%) of their healthcare workers’ sample exhibited pandemic-related burnout. Chor et al. [24] found that 53% of their sample were experiencing burnout, and Matsuo et al. [25] showed that 31.4% of a sample of healthcare workers from Japan were experiencing burnout. Denning et al. [26] showed that 67.1% of healthcare workers from a multinational cross-sectional study were positive for burnout, among others. Several studies have also analyzed the predictors on the relationship between the CoViD-19 pandemic and burnout, showing that work hours, work overload, being in close contact with people, anxiety symptoms, depression symptoms, emotional exhaustion, depersonalization, and insomnia are consistent predictors [26,27,28,29,30,31]. Furthermore, recent studies showed that other psychosocial risks such as social support, social value, being stigmatized as a healthcare worker, witnessing disease, lack of organizational justice, emotional work, distress intolerance, intolerance of uncertainty, interpersonal conflicts, and role conflicts are also significant predictors for burnout and mental distress [31,32,33,34].

Among psychosocial risks, one of the variables most extensively studied in the last year has been risk perception from CoViD-19. Several studies show that sociocultural and sociodemographic factors such as higher education level, prosocial values, and gender (females) are associated with higher risk perception. Having experience with the virus is also associated with higher risk perception [35,36]. Another critical finding regarding risk perception is that those with higher levels engage more in protective behaviors such as staying at home, hand washing, and disinfecting personal objects [37,38].

Recent studies have also given great importance to certain sociodemographic variables such as socioeconomic status (SE), strongly associated with the perceived severity of CoViD-19. Irigoyen-Camacho et al. (2020) exhibit that people with higher SE showed higher perceived severity and carried out more preventive actions. Multiple studies also showed that low-income individuals are more affected by the pandemic: household crowding and higher odds of working on-site are associated with higher CoViD-19 infection rates [37,39].

However, it is vital to know this impact on workers in other sectors to implement psychological prevention measures in these groups. The current pandemic has caused workers in continuous contact with other people to display changes in their daily tasks and in their work environment to adjust to the health prevention regulations. Also, environmental changes and performing uncommon tasks are well-known stressors correlated with burnout syndrome development [23].

In Spain, Oliver et al. [40] conducted a survey to quickly assess the Spanish citizens’ situation and perception on four areas related to the CoViD-19 pandemic: their social contact behavior during the confinement, their economic impact, their work situation, and their health status. They found a significant economic impact of the CoViD-19 pandemic in small businesses as 47.3% of respondents working in small (1–9 workers) companies reported having been financially affected, and 19.4% reported facing insolvency at their work. Fashion retailing was the third most affected in terms of occupations, only behind hospitality and construction. 9.3% of the participants who had or were in danger of losing their job/business were working in fashion retailing (only 5.3% of participants who did not think they will lose their jobs working in fashion retailing). 

As mentioned above, many studies analyzed the impact of the CoViD-19 on healthcare workers’ mental health; however, there is no research that we know on the pandemic’s impact on fashion’s retailing workers mental health. Therefore, the present study aims to evaluate the levels of mental workload and burnout manifested by fashion retailing workers in the context of CoViD-19 in Spain and determine which of the pandemic aspects are more determinant of the increase of mental workload and burnout. We expect that fashion retailing workers show similar results to mental health workers, such as similar predictors. It is necessary to highlight that it is difficult to find empirical studies analyzing the relationship between the CoViD-19 pandemic and higher levels of mental workload levels, which, in our opinion, constitutes a novel contribution to current literature. The present study also evaluates uncertainty and negative job expectations as possible predictors in the relationship of CoViD-19 pandemic and burnout and mental workload.

Lastly, it is necessary to highlight that it is difficult to find empirical studies distinguishing between psychosocial risks by jobs in this sector. Due to the diversity of the nature of each position’s tasks [41], the present study makes a distinction between job positions, which constitutes a novel contribution of this work to current literature.

## 2. Materials and Methods 

A cross-sectional design was established, which involved a random sample of 360 fashion retailing workers. Participants were recruited through LinkedIn and from disseminating this research through other social networks (i.e., Instagram, Twitter). It was necessary to be employed and to have at least six months seniority at the moment to participate. Those who wished to participate would access a link through a post or message that would lead to a Google Forms’ questionnaire. In the Google Forms’ questionnaire, they first gave their informed consent to participate in the study. We collected data throughout October and November 2020. The sample’s mean age was 32.48 years old, ranging from 19 to 56 years old, 31.7% were males and 68.3% were females. 

Participants answered an online survey that consisted of the following instruments:

Socio-demographical variables (Appendix A): Participants had to indicate their age, gender, educational level, civil status, if they have children or not, employment position if they have had any sick leave during the last year, cause of the sick leave, labor seniority, and type of contract.

CarMen-Q (Appendix B): Mental workload questionnaire (Cognitive Demands, Temporal Demands, Emotional Demands, Performance Demands) [42] was developed for the Spanish population and consisted of 29 multiple choice response items. Item’s response format was a Likert frequency scale of four alternatives in which 0 meant never, 1 rarely, 2 often, and 3 always. The authors stated the items so that a higher score indicated a higher mental workload. CarMen-Q questionnaire contained the following subscales: the factor “cognitive demands” consisted of 10 items related to the processing of complex information, difficulties in perceiving information, complex decision making, memory load, and the amount of information that needs to be taken into account to perform job tasks. The factor “temporal demands” was constituted by 7 items that ask for work rhythm, presence of annoying interruptions, or the possibility of taking breaks when the worker needs it. The factor “emotional demands” was formed by 7 items related to the job’s emotional and health consequences. Five items constituted the “performance demands” factor that asked about the performance requirements at the level of responsibility, required accuracy of responses, and error severity. In our sample, CarMen-Q had high reliability (Cronbach’ α = 0.93).

MBI (Appendix C): Burnout Syndrome Inventory (Emotional Exhaustion, Depersonalization, Personal Accomplishment) [43,44], which consisted of 22 multiple-choice response items, evaluated using a Likert scale with response options ranging from “never” (0) to “every day” (6). The questionnaire contained the following subscales: (1) Emotional Exhaustion, which assessed the experiences of being emotionally exhausted by the demands of work, (2) Depersonalization, which evaluated the degree to which the employee shows attitudes of coldness and detachment that occur in the workplace, (3) Personal Accomplishment, which assessed the feelings that the worker has of self-efficiency and fulfillment that occur in the workplace. MBI version for the Spanish population was used, and it met enough requirements for both factor validity and internal consistency [43,44]. In our sample, MBI had good reliability (Cronbach’ α = 0.82).

Perception of CoViD-19: participants answered questions related to how they perceived the CoViD-19 pandemic and how they thought it affects or has affected their job and their mental well-being. It consisted of 8 multiple choice response items, with a Likert scale from “totally disagree” (0) to “totally agree” (5). 

The Ethics Committee approved this study of the authors’ research center, obtaining a favorable report in September 2020 (Ref. 2019/20-022).

We performed all statistical analyses using IBM SPSS v. 25.0.

## 3. Results

First, we calculated descriptive statistics for all measures. 

Regarding sociodemographic data (Table 1), participants’ civil status results showed that 56.9% were single, 37.2% were married or had a stable partner, and 5.9% were separated, divorced, or widowed. Only 21.7% of participants had children. Concerning education level, 68.6% had university studies, 11.7% high school studies, 11.1% middle-grade studies, and 8.6% secondary school studies. It was necessary to have at least six month seniority at the moment to participate in this study: 19.7% had been on their companies between 1 and 2 years, 31.1% between 2 and 5 years, 21.7% between 6 months and 1 year, and 27.5% more than 5 years. Regarding job position, 41.7% were sales assistants, 26.4% store managers, 14.7% area managers, 6.9% assistant store managers, 3.1% heads of sales, 2.2% human resources technicians, 1.9% cashiers, 0.8% delivery drivers, 0.8% visual merchandising, 0.8% key account managers, and 0.6% marketing assistants. Regarding the type of contract, 78.9% had an indefinite contract, 20.8% had a fixed-term contract, and 0.3% were freelance. With respect to sick leaves, 32.8% had periods of sick leave in the last year, reasons were: CoViD-19 (11.4%), stress and/or anxiety (5.8%), musculoskeletal disorders (4.2%), gastric problems (2.5%), flu (1.9%), occupational accident (1.7%), surgery (1.7%), cardiovascular disorders (0.6%), pneumonia (0.6%), pregnancy (0.6%), cancer (0.3%), pharyngitis (0.3%), brain myelitis (0.3%), and traffic accident (0.3%). 

To explore the influence of the perception of CoViD-19 on mental workload and burnout, we computed seven multiple regression analyses, using the 8 items of the CoViD-19 questionnaire as predictors and each of the dimensions of workload and burnout as a dependent variable. To check the compliance of the non-multicollinearity assumption necessary to perform a multiple regression analysis, we calculated the tolerance and inflation factor of variance (VIF). In all cases, tolerance was greater than 0.10 (between 0.52 and 0.95) and IVF less than 10 (between 1.02 and 1.95), which indicated compliance with this assumption. The Durbin–Watson statistic reached values between 1.91 and 2.03, showing compliance with the assumption of independence of the errors, since they were greater than 1.5 and less than 2.5. Compliance with the homoscedasticity assumption was verified by observing the scatter plot of the standardized forecasts (ZPRED) and the standardized residuals (ZRESID). In all cases, compliance with the assumption of homoscedasticity was verified since no association patterns were seen. The value of the Kolmogorov–Smirnov statistic was not significant in all cases (*p* > 0.10), confirming compliance with the normality assumption of the standardized residuals and the dependent variables.

Table 2 shows the mean and standard deviation for all measures. The scores for the eight questions related to the influence of the current CoViD-19 pandemic denoted that participants exhibited deep concern about the CoViD-19 pandemic and its influence at the work level. It had created uncertainty in their jobs and some physical and psychological changes. Scores for the four mental workload dimensions were near the middle point of the scale (range from 0 to 3). Performance demands obtained the highest mean scores. Participants exhibited moderate to high levels of burnout. It is necessary to highlight that scores that denote the presence of high burnout were 26 or more for Emotional Exhaustion, 9 or more for Depersonalization, and 34 or less for Personal Accomplishment [45]. Thus, we can assume that our sample was at risk of presenting high burnout levels as long as the pandemic and its effects remain present.

Table 3 shows the results of multiple regression analysis. We obtained significant results for all dependent variables (*p* < 0.01). Cognitive Demands were significantly associated with the items “Now I work more than before the CoViD-19 pandemic” and “The work environment of my work is not the same since the CoViD-19 pandemic”, showing higher workload. Emotional Demands were significantly associated with several items, especially with “I have noticed physical changes (lack of sleep, muscle aches, irritability...) since the CoViD-19 pandemic”, “I fear that my work situation will be affected by the pandemic,” and “Now I work more than before the CoViD-19 pandemic”. Temporal Demands were significantly associated with the item “Now I work more than before the CoViD-19 pandemic”. Performance Requirements were significantly associated with the items “Now I work more than before the CoViD-19 pandemic” and “The work environment of my work is not the same since the CoViD-19 pandemic”. 

The results indicate that because of CoViD-19 there has been an increase in workload, which is associated with greater cognitive, emotional, temporal, and performance demands. Environmental changes, somatic symptoms, insomnia, negative job expectations, and uncertainty also constituted significant mental workload predictors. The participants’ perception of CoViD-19 influence on their jobs were most associated with Emotional Demands (*R*^2^ = 0.31) compared to other workload dimensions.

Emotional Exhaustion was the burnout dimension most associated with the participants’ perception of CoViD-19 (*R*^2^ = 0.25). Emotional Exhaustion was principally associated with the items “I have noticed physical changes (lack of sleep, muscle aches, irritability...) since the CoViD-19 pandemic” and “I think my work situation is going to get worse due to the CoViD-19 pandemic”. Depersonalization was significantly associated with the items “I have noticed physical changes (lack of sleep, muscle aches, irritability...) since the CoViD-19 pandemic”.

According to multiple regression analysis results, insomnia and somatic symptoms constituted a strong predictor for burnout. They were associated with the two subscales Emotional Exhaustion and Depersonalization from the MBI. In general, negative job expectations constituted a significant predictor for mental workload and burnout, especially regarding emotional issues.

To explore if there were statistical differences in mental workload and burnout due to several sociodemographic characteristics, we performed ANOVAs with marital status, job position, and gender as factors. No significant differences were found between married, separated, divorced or widowed, and single (*p* > 0.1 in all cases).

Regarding job position, we found differences between job positions for the subscale Personal Fulfillment from the MBI, which was significantly lower for sales assistants than store managers (I-J = −4.06) and area managers (I-J = −4.40). No differences between job positions were found for the subscales Emotional Fatigue and Depersonalization from the MBI. We found differences between job positions for the subscale Cognitive Demands from the CarMen-Q. Punctuations were significantly lower for sales assistants than for store managers (I-J = −0.56), heads of sales (I-J = −0.78), human resources technicians (I-J = −0.70), and area managers (I-J = −0.75). Secondly, area managers’ cognitive demands were significantly higher than those of human resources technicians (I-J = 0.44) and assistant store managers (I-J = 0.44). Differences between job positions were found for the subscale Performance Requirements from the CarMen-Q, being significantly lower for sales assistants than for store managers (I-J = −0.34) and area managers (I-J = −0.43) (see Table 4).

To explore the differences between men and women on burnout and mental workload, we computed a one-way ANOVA (Table 5). Comparison of the responses given by gender showed that women had higher levels of Emotional Exhaustion (men = 22.11; women = 27.65) (*p* ≤ 0.01) and higher levels of Emotional Demands (men = 1.25; women = 1.52) (*p* ≤ 0.01). On the other hand, men had higher levels of Cognitive Demands (men = 1.77; women = 1.51) (*p* ≤ 0.01) and Performance Requirements (men = 2.09; women = 1.88) (*p* ≤ 0.01). It is necessary to highlight that even though the differences were not significant for the three subscales, women met the criteria for severe burnout on Emotional Exhaustion (≤26).

Pearson correlation between age and mental workload and burnout were not significant except for the Cognitive Demands dimension of mental workload (r = 0.27, *p* < 0.01).

## 4. Discussion

The present study strongly suggests that the CoViD-19 pandemic affects fashion retailing employees’ mental workload and plays a vital role in the presence of burnout syndrome. The current concern about the pandemic, the negative future expectations about their job, the uncertainty it causes, and how it has changed employees’ daily work, physical well-being, and work environment, leads to higher mental workload levels and correlates with the presence of burnout syndrome. Even though the results showed that participants exhibit moderated mental workload levels and indicated that participants were at high risk of developing severe burnout syndrome shortly, these findings have important theoretical and practical implications. They point out that the CoViD-19 pandemic is negatively affecting fashion retailing employees’ mental well-being.

We found significant differences between job positions for some mental workload components. The exposure was higher in some hierarchically superior positions, such as store managers, area managers, and heads of sales, area managers being the most exposed to high cognitive demands. On the other hand, store managers and area managers were the most exposed to high-performance requirements. Also, we found significant differences between job positions for some components of burnout. The exposure was higher in some of the hierarchically lower positions, such as sales assistants: sales assistants had significantly lower personal fulfillment levels than store managers and area managers. Due to the lack of studies that differentiate between fashion retailing job positions, it has not been possible to compare our results. However, our results allow future research to better delve into this variable and its relationship with burnout and mental workload on fashion retailing workers in the pandemic’s current context.

Recent studies show that women’s emotional burnout levels are higher in the pandemic context since they suffer from a higher financial impact and are more susceptible to work–family role conflict [45,46,47,48,49,50,51,52,53,54,55,56,57,58]. Also, the emotional dimension of workload was more affected in women. Thus, our results are consistent with previous literature. 

Over the last year, several studies have analyzed healthcare workers’ mental workload levels during the current pandemic. However, the literature that evaluates the differences by gender in mental workload in the current pandemic context is scarce. These studies refer to the fact that women would be more affected in this dimension. Thus, our study results provide contrary conclusions: men working in fashion retailing had higher levels of mental workload’s cognitive and performance dimensions [59,60,61,62]. Therefore, we propose further research on gender differences on mental workload in fashion retailing to understand the differences between our study in fashion retailing workers and the existing literature on healthcare personnel.

Several studies this year have studied the impact of the CoViD-19 pandemic on healthcare workers’ mental health [62]. This study shows that the current pandemic directly impacts cognitive, emotional, performance, and temporal demands and the presence of burnout syndrome in workers who carry out their work in close contact with people. Thus, the case of customer-facing staff from fashion retailing is consistent with similar studies on healthcare workers [24,25,26,27,28]. According to current literature, somatic symptoms and insomnia constituted predictors of burnout [27,28,29,30,31,32]. However, environmental changes, work overload, uncertainty, and negative job expectations also constituted predictors for mental workload. These findings have a significant theoretical implication. This study points out that the uncertainty and negative future expectations about their job caused by the CoViD-19 pandemic can also predict burnout and mental workload. 

It is also necessary to highlight that this study points out that the emotional dimension was most affected by the participants’ perception of CoViD-19: emotional exhaustion and emotional demands were associated with insomnia and somatic symptoms, negative job expectations, uncertainty, and work overload. According to several studies, negative and passive emotions and emotional exhaustion predict lower in-role and extra-role performance [63], productivity loss [64,65], and lower work attitudes [66]. They are also associated with depression, anxiety, and impaired emotional functioning [27,67,68,69,70].

The present study has practical implications as well. One important recommendation for fashion retailing companies is to monitor the levels of mental workload and burnout in their employees and implement psychological prevention measures to avoid severe burnout syndrome or the increase to alarming levels of mental workload: (1) control of work organization, and the main elements that generate discomfort in the workforce, (2) involve employees in decision-making, (3) promote motivation and a positive work environment, (4) promote social support among the workforce, (5) avoid the perception of urgency and pressure on employees, (6) properly organize tasks, (7) provide some autonomy to workers, (8) avoid excesses or errors in the information transmitted to workers, (9) vary the levels of complexity in the tasks and (10) schedule enriching tasks. Also, another necessary recommendation for fashion retailing companies is to efficiently manage the negative job expectations of their workforce by (1) renegotiating their contracts in case of temporary closure, (2) ensure their permanence for a minimum period of 6 months after the completion of the Record of Temporary Employment Regulation (RTER), and (3) ensure the viability of a second RTER based on how sales evolve in the reopening phase. More efficient management of the psychological, physical, and environmental changes caused by the pandemic is essential since the exposure to these stressors has been remarkable throughout these months. Thus, another recommendation for companies is to improve the fit between the organization and the employees to better adapt to the work environment: (1) make existing tasks and new demands more flexible among the entire workforce, (2) make opening hours more flexible and (3) make working hours more flexible. Furthermore, fashion retailing companies should implement emotional protection measures: (1) encourage the management of emotions through training in relaxation techniques and handling stressful situations, (2) facilitate common spaces for communication, (3) promote motivation and a positive work environment, and (4) promote social support among the workforce. Lastly, to mitigate gender-based differences in burnout and job-concerns, we recommend retail companies to provide resources and flexibility in the workplace to women in order to increase their success: (1) flexible work hours, (2) family-friendly policies, (3) mentoring, and (4) peer support programs.

This study has limitations. Since we did not assess the baseline level of burnout before the pandemic, we could not compare prevalence changes. Further studies focusing on both identification and interventions for workers from sectors other than healthcare to prevent and reduce the risk of burnout are needed. Also, since we did not assess the baseline level of burnout on the first wave of the pandemic in Spain (March–June), we could not compare prevalence changes. As the first and second waves impacted differently on the country’s economy and health system, a comparative on burnout between both waves would have provided a clear and global vision of the CoViD-19 pandemic’s entire course in Spain. Even though we assessed participants’ health over the last months, we did not take information about the level of exposure to the virus in their environment (family and friends). As stress related to the pandemic seems to affect the experiences of work-related well-being, it would be essential to assess the participants’ health status, as well as their families’ and friends’. We propose further investigation assessing this variable. Another limitation found is that we did not assess the level of perceived stigma on participants. Recent studies [71] show that the stigma caused by being infected, in close contact with people diagnosed positive or at risk of contagion affects mental and medical health. Thus, we propose further investigation, including the assessment of this variable on Spanish fashion retailing workers. Also, we did not assess the socioeconomic status. As mentioned in the introduction, socioeconomic status is strongly associated with the perceived severity of CoViD-19. Also, individuals with low incomes are more affected by the pandemic. Thus, this variable could act as a mediating variable between the variables analyzed in this study and burnout. Evaluation of this variable in future research could also enrich our results [37,38]. Lastly, we did not assess the risk perception of CoViD-19 either. Recent research has shown the link between several sociocultural and sociodemographic variables (education level, gender, and values) and higher risk perception, which, in turn, is associated with preventive behaviors [35,36,37,38]. Thus, this variable could also act as a mediating variable between the variables analyzed in this study and burnout. Further investigation assessing this variable could as well enrich our results.

Throughout December 2020, the European Commission (EC) authorized vaccines from different laboratories to start vaccination against CoViD-19. On 21 December 2020, the EC authorized Pfizer’s and BioNTech’s vaccines. On January 6, 2021, the European Union authorized Moderna’s vaccine. Also, on 30 December 2020, the United Kingdom authorized Oxford-AstraZeneca’s vaccine. By the time we started gathering information for our study (October 2020), laboratories were still investigating their vaccines. Once laboratories reported that their vaccines had high efficacy and that they were waiting for authorization, anti-vaccine movements began to emerge with great strength. According to the WHO, vaccine hesitancy has been a global threat since 2019, since it “threatens to reverse progress made in tackling vaccine-preventable diseases” [72]. Due to the severity of the current pandemic, vaccine hesitancy has become a controversial topic and a severe problem for eradicating CoViD-19. Thus, further research assessing participants’ intentions regarding getting vaccinated will help better understand the population’s perception of the pandemic, such as their coping behaviors. 

## 5. Conclusions

Multiple studies have analyzed the effects of CoViD-19 on healthcare personnel’s mental health; however, there are no studies that analyze these effects in other professional groups that, although in a less direct way than healthcare workers, are also being affected by the pandemic. This study analyzed the levels of mental workload and burnout on Spanish fashion retailing workers in the current context caused by the CoViD-19 pandemic. This sector has great value in the Spanish economy, and most fashion retailing workers are in direct contact with people. In general, we found mean scores of mental workload near the middle point of the scale and moderate to high burnout levels, revealing that the sample was at risk of experiencing higher burnout levels over time as the pandemic and associated economic crisis continue. In this sense, it is convenient to gather more data in the following months to monitor these workers’ mental health.

Insomnia, somatic symptoms, job uncertainty, and negative job expectations constituted significant predictors for burnout and mental workload, especially to both constructs’ emotional dimensions (emotional exhaustion of burnout and emotional demands of the mental workload). As the main conclusion, the uncertainty at work derived from the CoViD-19 pandemic harms the psychological well-being of fashion retailing workers in Spain, like with healthcare personnel. So, fashion retail companies must monitor their employees’ mental workload and burnout levels and implement prevention strategies to avoid severe burnout syndrome or mental workload increase to alarming levels.

## Figures and Tables

**Table 1:** Characteristics of the participants.

Characteristic	Value	N	%
Gender	Female	246	68.3
	Male	114	31.7
Civil Status	Married/Stable partner	134	37.2
	Single	205	56.9
	Separated/Divorced/Widowed	21	5.9
Children	Yes	78	21.7
	No	282	78.3
Education Level	University Studies	247	68.6
	High School Studies	42	11.7
	Middle-Grade Studies	40	11.1
	Secondary Studies	31	8.6
Labor Seniority	Between 6 months and 1 year	78	21.7
	Between 1 and 2 years	71	19.7
	Between 2 and 5 years	112	31.1
	More than 5 years	99	27.5
Job position	Sales Assistant	150	41.7
	Store Manager	95	26.4
	Area Manager	53	14.7
	Assistant Store Manager	25	6.9
	Head of Sales	11	3.1
	Human Resources Technician	8	2.2
	Cashiers	7	1.9
	Delivery Drivers	3	0.8
	Visual Merchandiser	3	0.8
	Key Account Manager	3	0.8
	Marketing Assistant	2	0.6
Sick leave	Yes	118	67.2
	No	242	32.8
Sick leave cause	CoViD-19	41	11.4
	Stress or Anxiety	21	5.8
	Musculoskeletal Disorders	15	4.2
	Gastric Problems	9	2.5
	Occupational Accident	6	1.7
	Cancer	1	0.3
	Cardiovascular Disorders	2	0.6
	Other	23	6.3

**Table 2:** Mean, standard deviation (SD) and median for all measures.

Measure	Item/Variable	Mean	SD	Median
CoViD-19	I am not worried about the CoViD-19 pandemic	1.74	1.25	1.00
	The CoViD-19 pandemic creates uncertainty in my job	3.99	1.35	4.30
	I have noticed physical changes (lack of sleep, muscle aches, irritability...) since the CoViD-19 pandemic	3.46	1.49	3.90
	I fear that my work situation will be affected by the pandemic	4.25	1.15	4.60
	The CoViD-19 pandemic has affected several aspects of my work activity	4.46	0.95	4.50
	Now I work more than before the CoViD-19 pandemic	3.04	1.50	3.00
	The work environment of my work is not the same since the CoViD-19 pandemic	3.74	1.35	3.90
	I think my work situation is going to get worse due to the CoViD-19 pandemic	3.88	1.26	4.00
Mental workload	Cognitive Demands	1.59	0.63	1.65
	Emotional Demands	1.44	0.74	1.43
	Temporal Demands	1.59	0.66	1.57
	Performance Demands	1.94	0.61	2.00
Burnout	Emotional Exhaustion	25.90	12.36	25.00
	Depersonalization	8.06	6.20	7.00
	Personal Accomplishment	35.45	6.98	36.00

**Table 3:** Results of multiple regression analysis of CoViD-19 perceptions on each dimension of mental workload and burnout.

Item	Cognitive Demands		Emotional Demands		Temporal Demands		Performance Demands		Emotional Exhaustion		Depersonalization		Personal Accomplishment	
	*β*	*p*	*β*	*p*	*β*	*p*	*β*	*p*	*β*	*p*	*β*	*p*	*β*	*p*
I am not worried about the CoViD-19 pandemic	0.04	0.47	−0.04	0.43	−0.03	0.50	0.01	0.91	0.03	0.46	0.08	0.13	0.02	0.74
The CoViD-19 pandemic creates uncertainty in my job	0.05	0.40	0.11	0.03	0.11	0.06	0.16	0.01	0.04	0.45	−0.02	0.74	−0.05	0.47
I have noticed physical changes (lack of sleep, muscle aches, irritability...) since the CoViD-19 pandemic	−0.01	0.86	0.34	0.00	0.07	0.22	−0.07	0.24	0.37	0.00	0.28	0.00	−0.14	0.02
I fear that my work situation will be affected by the pandemic	0.12	0.09	0.27	0.00	0.15	0.03	0.10	0.16	0.22	0.00	0.21	0.00	0.08	0.26
The CoViD-19 pandemic has affected several aspects of my work activity	0.02	0.72	−0.01	0.92	0.02	0.74	0.04	0.49	−0.01	0.82	−0.03	0.62	0.11	0.10
Now I work more than before the CoViD-19 pandemic	0.26	0.00	0.19	0.00	0.35	0.00	0.19	0.00	0.14	0.00	0.07	0.20	0.11	0.04
The work environment of my work is not the same since the CoViD-19 pandemic	0.20	0.00	0.16	0.00	0.09	0.12	0.21	0.00	0.02	0.71	0.03	0.65	0.07	0.25
I think my work situation is going to get worse due to the CoViD-19 pandemic	−0.05	0.44	0.16	0.01	0.07	0.31	−0.02	0.72	0.24	0.00	0.19	0.01	−0.16	0.02
*R*	0.36		0.56		0.43		0.33		0.50		0.35		0.22	
*R^2^*	0.13		0.31		0.19		0.11		0.25		0.12		0.05	
*F*	6.55		19.95		10.08		5.37		14.58		6.03		2.17	

**Table 4:** Results of comparison between job positions.

Variable	(I) Job Position	(J) Job Position	Means Differences (I-J)	Sig.
Personal Fulfillment	Sales assistant	Store manager	−4.06	0.000
		Area manager	−4.40	0.003
	Store manager	Sales assistant	4.06	0.000
	Area manager	Sales assistant	4.40	0.003
Cognitive Demands	Sales assistant	Store manager	−0.56	0.000
		Head of sales	−0.78	0.000
		Area manager	−0.75	0.000
	Store manager	Sales assistant	0.56	0.000
	Head of sales	Sales assistant	0.78	0.000
	Area manager	Sales assistant	0.75	0.000
Performance Requirements	Sales assistant	Store manager	−0.34	0.001
		Area manager	−0.43	0.000
	Store manager	Sales assistant	0.34	0.001
	Area manager	Sales assistant	0.43	0.000

**Table 5:** Results of comparisons between men and women.

Variable	Gender	Mean	*SD*	*T*	*p*
Cognitive Demands	Female	1.51	0.63	13.23	0.000
	Male	1.77	0.60		
Emotional Demands	Female	1.52	0.77	10.80	0.001
	Male	1.25	0.65		
Performance Requirements	Female	1.88	0.63	9.36	0.002
	Male	2.09	0.54		
Emotional Exhaustion	Female	27.65	12.74	16.32	0.000
	Male	22.11	10.62		

## Data Availability

The data presented in this study are available on request from the corresponding author.

## Appendix A

### Socio-Demographical Variables

1. Job position (open question)
2. Age (open question)
3. Gender
  (a) Male
  (b) Female
  (c) Other
4. Level of studies
  (a) University Studies
  (b) High School Studies
  (c) Middle-Grade Studies
  (d) Secondary Studies
5. Civil status
  (a) Single
  (b) Married / Stable partner
  (c) Separated / Divorced / Widowed
6. Do you have children?
  (a) Yes
  (b) No
7. How long have you been working at your current company?
  (a) Between 6 months and 1 year
  (b) Between 1 and 2 years
  (c) Between 2 and 5 years
  (d) More than 5 years
8. Type of contract you currently have.
  (a) Fixed-term
  (b) Indefinite
  (c) Other
9. Have you suffered a sick leave in the last twelve months?
  (a) Yes
  (b) No

If so, indicate the reason for the absence from work:

(a) Stress or anxiety
(b) Depression
(c) CoViD-19
(d) Cardiovascular disorders
(e) Musculoskeletal disorders
(f) Occupational accident
(g) Carcinogenic processes
(h) Gastric problems
(i) Others

## Appendix B

### CarMen-Q. Mental Workload Questionnaire

This questionnaire is evaluated using a Likert scale with response options ranging from “never” (0) to “always (3), therefore, the response options to all questions are (0) never, (1) sometimes, (2) often or (3) always.

1. My job requires maintaining a high level of attention (Mi trabajo requiere mantener un elevado nivel de atención)
2. My work involves the processing of complex information (Mi trabajo implica el tratamiento de información compleja)
3. My job requires thinking and choosing between different alternatives (Mi trabajo requiere pensar y elegir entre diferentes alternativas)
4. I have to make diffi cult decisions (Tengo que tomar decisiones difíciles)
5. My job requires handling a lot of knowledge(Mi trabajo requiere manejar muchos conocimientos)
6. I have to work constantly, I cannot take breaks beyond strict regulations (Tengo que trabajar constantemente, no puedo hacer pausas, más allá de las estrictamente reglamentarias)
7. The pace of work is excessive, diffi cult to reach even by an experienced worker(El ritmo de trabajo es excesivo, difícil de alcanzar incluso por un trabajador experimentado)
8. I often work with annoying interruptions(Suelo trabajar con interrupciones molestas)
9. I cannot stop my work when I need it (No puedo parar o detener mi trabajo cuando lo necesito)
10. The pace of work is imposed on me (El ritmo de trabajo me viene impuesto)
11. The accomplishment of my tasks demands a lot of speed (La realización de mis tareas exige mucha rapidez)
12. It is normal for me to accumulate the tasks (Es normal que se me acumulen las tareas)
13. My job requires no mistakes (Mi trabajo requiere que no se cometa ningún error)
14. I have to give very precise responses (Tengo que dar respuestas muy precisas)
15. My mistakes can have serious consequences (Mis errores pueden tener consecuencias graves)
16. My job requires dealing with information that is perceived with diffi culty (Mi trabajo requiere tratar con información que se percibe con difi cultad)
17. I have trouble forgetting the problems of my job (Me cuesta olvidar los problemas de mi trabajo)
18. My work makes me nervous (Mi trabajo me pone nervioso)
19. My work is affecting my personal relationships (family, friends...) (Mi trabajo está afectando a mis relaciones personales (familia, amigos..))
20. My job involves a lot of responsibility (Mi trabajo implica mucha responsabilidad)
21. I feel very tired, physically fatigued (Me siento muy cansado, fatigado físicamente)
22. I have to deal with information that is not easily understood (Tengo que tratar con información que no se entiende fácilmente)
23. My job requires a lot of information (Mi trabajo requiere el tratamiento de gran cantidad de información)
24. My work affects me a lot emotionally (Mi trabajo me afecta mucho emocionalmente)
25. My job requires memorizing a high amount of data (Mi trabajo requiere memorizar una cantidad elevada de datos)
26. My work is mentally intense (Mi trabajo es mentalmente intenso)
27. I have to do a great search and information gathering to carry out my tasks (He de realizar una gran búsqueda y recopilación de información para llevar a cabo mis tareas)
28. When I fi nish my workday I feel a lot of physical exhaustion (Al terminar mi jornada laboral siento mucho agotamiento físico)
29. My work is affecting my health (Mi trabajo está afectando a mi salud)

## Appendix C

### MBI: Maslach Burnout Inventory

Indicate how frequently the following statements apply to you and add the points indicated on top of the respective box:

0 = Never

1 = At least a few times a year

2 = At least once a month

3 = Several times a month

4 = Once a week

5 = Several times a week

6 = Every day

I feel emotionally exhausted because of my work

I feel worn out at the end of a working day

I feel tired as soon as I get up in the morning and see a new working day stretched out in front of me

I can easily understand the actions of my colleagues/supervisors

I get the feeling that I treat some clients/colleagues impersonally, as if they were objects

Working with people the whole day is stressful for me

I deal with other people’s problems successfully

I feel burned out because of my work

I feel that I influence other people positively through my work

I have become more callous to people since I have started doing this job

I’m afraid that my work makes me emotionally harder

I feel full of energy

I feel frustrated by my work

I get the feeling that I work too hard

I’m not really interested in what is going on with many of my colleagues

Being in direct contact with people at work is too stressful

I find it easy to build a relaxed atmosphere in my working environment

I feel stimulated when I been working closely with my colleagues

I have achieved many rewarding objectives in my work

I feel as if I’m at my wits’ end

In my work I am very relaxed when dealing with emotional problems

I have the feeling that my colleagues blame me for some of their problems
